# Lipopolysaccharide architecture dictates susceptibility to predation by *Bdellovibrio bacteriovorus*

**DOI:** 10.1093/ismejo/wrag239

**Published:** 2026-09-14

**Authors:** Hyeon Seop Lee, Sumin Choi, Wonsik Mun, Robert J Mitchell

**Affiliations:** Department of Biological Sciences, Ulsan National Institute of Science and Technology, 50 UNIST-gil, Eonyang-eup, Ulsan 44919, South Korea; Department of Biological Sciences, Ulsan National Institute of Science and Technology, 50 UNIST-gil, Eonyang-eup, Ulsan 44919, South Korea; Department of Biological Sciences, Ulsan National Institute of Science and Technology, 50 UNIST-gil, Eonyang-eup, Ulsan 44919, South Korea; Department of Biological Sciences, Ulsan National Institute of Science and Technology, 50 UNIST-gil, Eonyang-eup, Ulsan 44919, South Korea

**Keywords:** *Bdellovibrio bacteriovorus*, lipopolysaccharide, predation, O-antigen

## Abstract

Predatory bacteria such as *Bdellovibrio bacteriovorus* exert strong top–down control on Gram-negative bacterial populations, yet the prey surface determinants governing susceptibility to predation remain incompletely resolved. Lipopolysaccharide (LPS) is a dominant structural component of the Gram-negative outer membrane and has long been implicated in predator attachment and invasion, but how specific features of LPS architecture influence predation efficiency remains unclear. Here, the susceptibility of *Escherichia coli* K-12 mutants bearing defined truncations in LPS core biosynthesis was systematically examined using *B. bacteriovorus* 109J and three additional *Bdellovibrio*, HD100, BD-DH1, and EY2.3. Disruption of inner core biosynthesis genes (*waaC*, *waaD*, *waaE*, and *waaF*) or the *waaG* gene significantly delayed predation by all four predators. Reduced susceptibility scaled with the extent of truncation, with the shortest LPS structures conferring the greatest reduced susceptibility although none of the mutants were completely resistant and long-term killing was observed. In contrast, susceptibility to outer core truncations varied among predators, revealing strain-specific predation patterns. Restoration of O-antigen biosynthesis reduced prey susceptibility to some predators, consistent with the possibility that O-antigen sterically masks LPS-associated surface features involved in predation. Beyond its structural role, LPS also modulated predator activity. Whereas exogenous LPS acted as a decoy and delayed predation when added concomitantly with predators, pre-exposures accelerated prey killing, indicating extracellular LPS modulates predation activities and rates. Together, these findings demonstrate that the prey LPS architecture plays an important role in shaping *Bdellovibrio* predatory activities, with implications for predator–prey dynamics in Gram-negative microbial communities.

## Introduction

Predatory bacteria are increasingly recognized as important regulators of microbial community structure, contributing to nutrient cycling, population turnover, and the maintenance of diversity in complex ecosystems [1]. Among these organisms, *Bdellovibrio bacteriovorus* and related obligate predators are distinguished by their ability to actively invade and kill Gram-negative bacteria. Through their predatory lifestyles, *Bdellovibrio*-and-like organisms (BALOs) influence bacterial abundance in aquatic, soil, and host-associated environments and have attracted interest as potential biological control agents against pathogenic bacteria [2–6].

Successful predation by *B. bacteriovorus* requires a tightly coordinated sequence of events that includes prey recognition, attachment, invasion of the periplasm, and formation of a bdelloplast in which the predator grows and replicates [7]. Each step depends on intimate interactions between the predator and the prey cell envelope. Previous work has identified several prey surface features that influence susceptibility, including outer membrane proteins and surface appendages [8–10]. However, the relative importance of these features and how they are integrated during prey recognition remain unresolved.

Lipopolysaccharide (LPS) is a defining component of the Gram-negative outer membrane and represents a particularly attractive candidate for mediating predator–prey interactions. LPS consists of lipid A, a core oligosaccharide, and, in many bacteria, an O-antigen polysaccharide [11, 12]. The inner core region is highly conserved and structurally constrained, whereas the outer core and O-antigen are more variable and subject to environmental and evolutionary pressures [13]. Variation in LPS architecture is known to profoundly influence interactions with bacteriophages [14–18], antimicrobial peptides [19], and host immune systems [20], yet its role in bacterial predation remains comparatively underexplored.

Early studies demonstrated gross disruption of prey envelope/LPS features can impair attachment of *B. bacteriovorus* to prey cells [21, 22], suggesting that intact LPS is required for efficient predation [23]. More recent work has implicated specific prey envelope components, specifically outer membrane porins and related proteins [9], in facilitating invasion. Nevertheless, a systematic evaluation of how defined alterations in LPS architecture affect susceptibility to *B. bacteriovorus* predation is lacking. For instance, it remains unclear whether reduced susceptibility arises from loss of specific recognition motifs, from physical shielding of the prey surface, or from broader changes to membrane organization.

In addition to its structural role, LPS may also influence predator physiology more directly. Attack-phase *B. bacteriovorus* cells, although obligately predatory, are capable of limited metabolic activity in the absence of prey [24] and can respond to extracellular nutrients [25]. Whether prey-derived LPS can serve as a metabolic cue or energy source that modulates predation efficiency has not been examined.

This study addresses these gaps by systematically dissecting the contribution of LPS architecture to prey susceptibility using a panel of *Escherichia coli* K-12 mutants with defined truncations in LPS core biosynthesis. By comparing predation by four *B. bacteriovorus* strains with distinct predatory phenotypes and by examining the effects of exogenous LPS on predator killing efficiency, we identify a constrained LPS architectural window, a “Goldilocks” zone, that optimally supports predation. These findings provide mechanistic insight into prey recognition and situate LPS-mediated susceptibility within a broader ecological framework of predator–prey interactions.

## Materials and methods

### Bacterial strains and culturing conditions

All bacterial strains used in this study are listed in Table S1. Prey strains and their corresponding isogenic mutants were routinely maintained on lysogeny broth (LB) agar plates supplemented with the appropriate antibiotics when required. For each experiment, fresh single colonies were inoculated into LB and incubated overnight at 37°C with shaking. Before the predation assays, the overnight cultures were harvested and washed three times with dilute nutrient broth [DNB; 1/10 nutrient broth (NB)] supplemented with 3 mM MgCl₂ and 2 mM CaCl₂ to remove residual antibiotics. Predatory bacterial strains were routinely grown as described previously using *E. coli* MG1655 as the prey strain [26, 27].

### Bioluminescence-based assay to quantify reduced susceptibility of LPS mutants to predation

*Escherichia coli* BW25113 and its isogenic Keio collection mutants [28, 29] were rendered bioluminescent by transformation with pGenluxCDABE, a gift from Harry Mobley (Addgene plasmid #44918; RRID: Addgene_44 918) [9, 30]. Overnight cultures were grown in LB broth supplemented with ampicillin (100 μg/ml) at 37°C with shaking. The cultures were then diluted to an OD_600_ of 0.1 in DNB supplemented with 3 mM MgCl₂ and 2 mM CaCl₂.

The predatory bacterial strains, *B. bacteriovorus* 109J, HD100, BD-DH1, and EY2.3 (Table S1), were propagated as described previously [9, 26, 27]. Predator suspensions were passed through 0.45-μm PES membrane filters (Millipore, USA) to remove residual prey cells prior to use in predation assays.

For the bioluminescence assays, the predator was diluted 1:4 in DNB supplemented with 3 mM MgCl₂ and 2 mM CaCl₂, and prey suspensions were mixed at a 1:1 volume ratio by adding 100 μl of each suspension to white 96-well microplates (Greiner, USA). The bioluminescence was measured at 10-min intervals for 90 min. The relative bioluminescence is defined as the bioluminescence of the test condition (predated *E. coli*) divided by that of the unpredated control at the same time point (Relative bioluminescence = BL_Predated_/BL_Control_ at the same time point). The initial predator and prey densities were determined by plaque enumeration using a top-agar overlay assay and by colony counting, respectively. These values were used to calculate the predator-to-prey ratio (PPR) for each assay.

### Long-term predation assays using *E. coli* LPS mutants

Predation assays were performed using *E. coli* BW25113 and its LPS mutant derivatives, including Δ*waaC*, Δ*waaF*, Δ*waaG*, Δ*waaI*, and Δ*waaL* [28, 29], as prey strains. The prey strains were grown overnight in LB broth supplemented with kanamycin (25 μg/ml) when required. The overnight cultures were harvested and resuspended in 15 ml of DNB to an OD_600_ of 1.0, corresponding to ~5 × 10^8^ colony-forming unit (CFU)/ml.

For these tests, 109J was prepared from an overnight predatory culture and passed through a 0.45-μm membrane filter to remove residual prey cells. Predation was initiated by adding 100 μl of the filtered predator suspension to each prey suspension, resulting in a final predator concentration of ~1 × 10^7^ plaque-forming unit (PFU)/ml (PPR 0.02). To assess the effect of LPS mutations on long-term susceptibility to predation, viable prey cells were quantified by CFU enumeration at 0 and 24 h.

### Confocal microscopy–based quantification of predator attachment and bdelloplast formation

A fluorescent 109J strain was generated by conjugative transfer of the tdTomato-expressing plasmid pMQ414 [31, 32] from *E. coli* S17 λ-pir into this predator. For plasmid maintenance, 109J carrying pMQ414 was cultured in medium supplemented with gentamicin at a final concentration of 10 μg/ml. The fluorescent predator was propagated as described above, concentrated 10-fold by centrifugation at 10 000 × *g* for 15 min, and resuspended in fresh 4-(2-hydroxyethyl)-1-piperazineethanesulfonic acid (HEPES) buffer (25 mM HEPES containing 3 mM MgCl₂ and 2 mM CaCl₂, pH 7.4).

Wild-type *E. coli* BW25113 and its isogenic LPS mutants, Δ*waaC*, Δ*waaF*, Δ*waaG*, Δ*waaI*, and Δ*waaL*, were used in parallel as prey strains. Each prey strain was grown overnight as described above, harvested by centrifugation at 7000 × *g* for 15 min, and resuspended in sterile HEPES buffer to an OD_600_ of 4.0. Predator and prey suspensions were mixed at a 1:1 volume ratio and incubated at 30°C with shaking. Samples were collected at 20 and 60 min after mixing. Each sample was immediately fixed by adding an equal volume of 8% (w/v) paraformaldehyde prepared in HEPES buffer, resulting in a final paraformaldehyde concentration of 4% (w/v). These fixed samples were stored at 4°C until imaging.

Fixed samples were mounted on glass slides, gently covered with coverslips, and observed using a confocal laser-scanning microscope (LSM780 Configuration 16 NLO, Carl Zeiss) equipped with transmitted-light differential interference contrast (DIC) optics. 109J carrying pMQ414 was detected based on its tdTomato fluorescence. The DIC images were acquired separately from the same microscopic fields using transmitted-light illumination. The tdTomato fluorescence and DIC channels were then merged only for visualization using ZEN software.

At each time point, prey cells were classified into three categories: free prey cells, prey cells with attached predators, and bdelloplasts. Cells in each category were manually counted to quantify predator attachment and bdelloplast formation during predation.

### Effect of LPS pretreatment on predation by *B. bacteriovorus* 109J

To examine the effect of exogenous LPS on predation by 109J, purified LPS from *E. coli* K-12 (LPS-EK standard; InvivoGen) [33] was used. LPS was dissolved in sterile 25 mM HEPES buffer to a stock concentration of 400 μg/ml. An overnight culture of predatory 109J was filtered to remove residual prey cells and diluted in sterile 25 mM HEPES buffer to achieve a predator density of 1 × 10^8^ PFU/ml.

Several conditions were explored in parallel, including co-treatment tests. These tests were performed by preincubating 109J in HEPES buffer (5 × 10^7^ PFU/ml) for 1 h at 30°C with shaking. Afterwards, the predator (200 μl), LPS (100 μl; 400 μg/ml stock), and prey (15 ml, OD 1.0 in HEPES) were mixed together and the tube was incubated at 30°C with shaking. The final LPS concentration (2.6 μg/ml) in these tests corresponds to that present in ~1.3 × 10^8^  *E. coli* cells using the Harvard BioNumbers database value of 3.46 × 10^6^ LPS molecules per *E. coli* cell (BNID 100076) [34]. Predation was monitored by measuring the OD_600_ and viable prey counts at 0, 9, 12, 15, 18, and 21 h. Viable prey cells were quantified and expressed as CFU/ml.

In parallel, we also tested pretreatments on predation rates. For these tests, LPS-free, LPS-pretreatment, NB-pretreatment, and NB plus LPS-pretreatment were all explored. Briefly, 100 μl of 109J was mixed with 100 μl of either HEPES, LPS (400 μg/ml stock), NB (1×), or LPS (400 μg/ml) suspended in NB and these samples were incubated for 1 h at 30°C with shaking. Subsequently, the entire sample was added to the prepared prey suspension (15 ml). Predation was monitored by measuring the OD_600_ and viable prey counts at 0, 9, 12, 15, 18, and 21 h. Viable prey cells were quantified and expressed as CFU/ml.

### Assessing LPS-mediated killing of *E. coli* BW25113

To evaluate the potential antibacterial activity of LPS against *E. coli* BW25113 in the absence of 109J, the prey cells were harvested and resuspended in sterile 25 mM HEPES to an OD_600_ of 1.0. A 200 μl aliquot of the LPS solution prepared at the same test concentration (200 μg/ml) was incubated for 1 h under the same conditions as above and an equal volume of the prepared *E. coli* suspension was added. As a control, HEPES buffer without LPS was used. The samples were then incubated for 24 h, and the bacterial viabilities assessed by serial dilution and CFU enumeration on LB agar plates.

### Competition-based predation assay using mixed prey cultures

To evaluate the effects of LPS biosynthesis mutations on prey susceptibility to 109J, *E. coli* BW25113 and its isogenic LPS mutant strains, Δ*waaC*, Δ*waaF*, Δ*waaG*, and Δ*waaL*, were used as prey. Each *E. coli* strain was grown overnight in LB broth at 37°C with shaking. 109J was also grown overnight as described above, passed through a 0.45-μm membrane filter to remove residual prey cells and cell debris, and diluted 1:4 in DNB.

Each *E. coli* strain was adjusted in DNB to an OD_600_ of 0.05, corresponding to ~2.5 × 10^7^ CFU/ml, and mixed with wild-type BW25113 at a 1:1 volume ratio to generate the mixed prey cultures. These prey suspensions were then mixed with 109J suspensions at a 1:1 volume ratio and incubated at 30°C with shaking for 90 min to allow predation to occur. At 90 min, the prey viabilities were assessed by CFU enumeration. Each sample was serially diluted in sterile DNB and plated on nonselective LB agar to determine the total number of surviving prey cells (both wild-type and isogenic mutant). In parallel, the same dilutions were plated on LB agar supplemented with kanamycin at 25 μg/ml to selectively enumerate the kanamycin-resistant LPS mutant strains. Each plate was incubated at 37°C, and the colonies were counted after incubation overnight to determine the population of each.

### Mutant complementation

Complementation of the LPS core biosynthesis mutants was performed using the ASKA clone library [35]. ASKA strains harboring pCA24N-derived plasmids carrying the corresponding wild-type genes, *waaC*, *waaF*, or *waaG*, were grown overnight in LB broth supplemented with 30 μg/ml chloramphenicol at 37°C with shaking. The individual plasmids were isolated from overnight cultures using a plasmid mini-prep kit according to the manufacturer’s instructions.

The purified plasmids, pCA24N::*waaC*, pCA24N::*waaF*, and pCA24N::*waaG*, were introduced into their corresponding *E. coli* BW25113 isogenic mutants by electroporation, and transformants were selected on LB agar plates supplemented with 30 μg/ml chloramphenicol. In parallel, wild-type *E. coli* BW25113 carrying the empty pCA24N vector was prepared and used as a vector control. The resulting complemented strains and the vector-control strain were used in subsequent predation assays as described below.

### Predation assay of ASKA-complemented LPS core mutants

Predation assays were performed to determine whether complementation of LPS core biosynthesis genes restored the susceptibility of the different *E. coli* LPS mutants to predation by 109J. The prey strains used in this assay included wild-type *E. coli* BW25113, the uncomplemented LPS core mutants Δ*waaC*, Δ*waaF*, and Δ*waaG*, wild-type BW25113 carrying the empty pCA24N vector, and the corresponding complemented mutants carrying pCA24N::*waaC*, pCA24N::*waaF*, or pCA24N::*waaG*. These prey strains were grown overnight in LB broth at 37°C with shaking in the presence of the appropriate antibiotics. Overnight cultures were harvested as described above, washed with fresh DNB, and adjusted in the same to an OD_600_ of 0.1.

109J was propagated as described above, passed through a 0.45-μm membrane filter to remove residual prey cells, and diluted 1:4 in fresh DNB. Predation assays were then initiated by mixing the prey and predator suspensions at a 1:1 volume ratio. The mixtures were incubated at 30°C with shaking for 90 min, after which the prey viabilities were assessed by serial dilution and CFU enumeration on LB agar plates with appropriate antibiotics. Wild-type BW25113 and BW25113 carrying the empty pCA24N vector were included as reference controls to assess the effect of vector carriage on predation susceptibility.

### Assessment of O-antigen contribution to predation activity

To assess the contribution of O-antigen biosynthesis on predation susceptibility, *E. coli* BW25113 was transformed with pMF19, a plasmid carrying a functional *wbbL* gene that enables O-antigen biosynthesis in the *E. coli* K-12 background [36, 37]. Plasmid pMF19 was obtained from Miguel Valvano (Addgene plasmid #113662; RRID: Addgene_113 662). A Δ*waaL* isogenic mutant carrying pMF19 was included as a negative control to evaluate the effects when O-antigen ligation is impaired (Fig. 1A).

**Figure 1:**
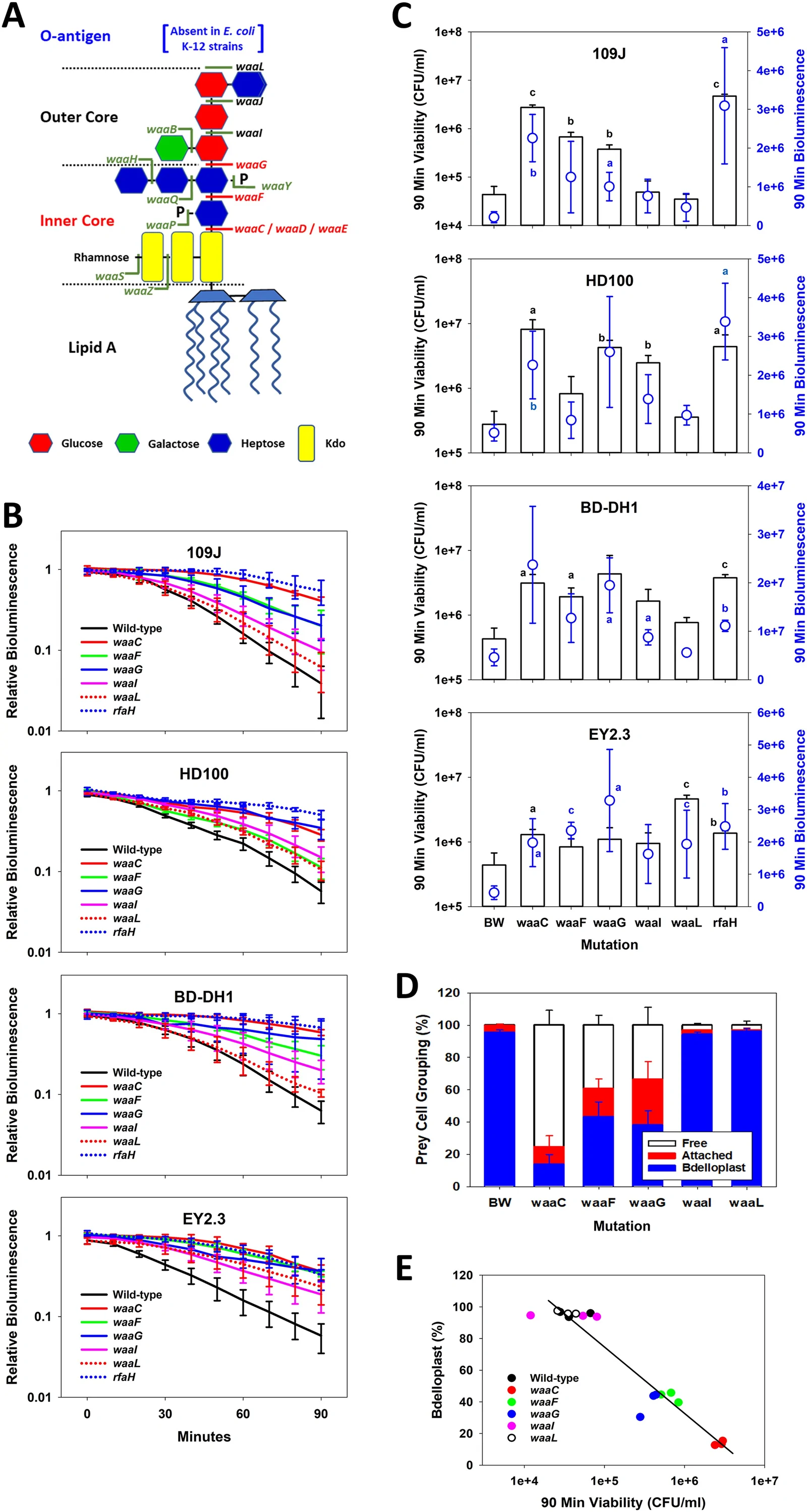
Impact of LPS truncation on susceptibility of *E. coli* to *B. bacteriovorus*. (A) Schematic of the *E. coli* K-12 LPS structure highlighting the genes deleted in this study. Inner core, outer core, and O-antigen regions are indicated, with corresponding biosynthetic genes annotated. (B) Prey bioluminescence following 1.5 h co-incubation of the indicated LPS truncation mutants with four different *B. bacteriovorus* strains (109J, HD100, BD-DH1, and EY2.3). The measured PPR for each sample was between 6 and 15. The relative bioluminescence is defined as the BL_Predated_/BL_Control_ at the same time point. (C) Predation outcomes of four *Bdellovibrio* strains, 109J, HD100, BD-DH1, and EY2.3, were assessed against wild-type *E. coli* BW25113 and LPS biosynthesis mutants after 90 min of co-incubation. Prey cell viabilities were determined by CFU enumeration, shown as bars on the left *y*-axis, and bioluminescence value, shown as circles on the right *y*-axis. The PPR for each sample was ~7. Statistics between the wild-type (BW) and isogenic mutants: ns, not significantly different; b, *P <* .01; d, *P <* .0001; *n* = 3. (D) microscopy-based classification of prey cell fates after 1 h of predation by tdTomato-expressing 109J. Cells were scored as free prey, predator-attached prey, or bdelloplasts. The PPR for each sample was ~7. *n* > 70 for each test. (E) correlation between the log_10_ reduction in viability and percentage of bdelloplast formation across several LPS mutants. The strong correlation (*R*^2^ = 0.84) indicates bdelloplast formation predicts killing efficiency.

Each prey strain was grown overnight in LB broth supplemented with 50 μg/ml spectinomycin, washed, and adjusted to an OD_600_ of 0.1 in DNB. Predatory strains, including *B. bacteriovorus* 109J, HD100, BD-DH1, and EY2.3, were prepared as described above, filtered through a 0.45-μm membrane filter, and diluted 1:4 in DNB.

For predation assays, the predator and prey suspensions were mixed at a 1:1 volume ratio (100 μl each) in 96-well cell culture plates (SPL, Korea), resulting in PPRs of ~10. These plates were incubated at 30°C with shaking, and the viable prey cells were quantified by serial dilution and CFU enumeration at 1 and 3 h, with these values expressed as CFU/ml.

### Synchronized attack protocol

Samples for western blot analysis were prepared using a synchronized predation assay. Briefly, overnight cultures of the prey (*E. coli* BW25113) were harvested by centrifugation (8000 × *g*, 10 min) and concentrated to an OD_600_ of 9.0*.* 109J were concentrated to 1 × 10^10^ PFU/ml by centrifugation (15 000 × *g*, 20 min) and resuspended in HEPES buffer. The prey and predator suspensions were mixed at a 1:1 (v/v) ratio to initiate the predation assay and incubated at 30°C shaking incubator at 250 rpm. Samples were collected immediately after mixing to determine the initial prey CFUs and predator PFUs. The prey cell viability was further assessed 15 min post-inoculation.

### Western blotting

Following synchronized predation, samples were collected at defined time points (0, 15, 30, 60, 90, 120, and 180 min). The bacteria were lysed by mixing the samples with 2× Sodium Dodecyl Sulfate (SDS) loading buffer [1:1 (v/v)] and heated at 70°C for 5 min to preserve LPS epitopes. Prepared lysates were stored at −80°C until further analysis. Samples were resolved on 4%–20% precast polyacrylamide gels (Bio-Rad) and transferred onto Immobilon-PSQ polyvinylidene fluoride (PVDF) membranes (Millipore) at 15 V for 30 min using a Tris-glycine SDS (48 mM Tris, 39 mM glycine, 0.037% SDS) transfer buffer with 20% methanol.

The PVDF membrane was directly blocked using a buffer consisting of 5% skim milk in Tris-buffered saline (TBS) for 1 h. After three washes with TBS for 5 min each, the membrane was incubated with the primary antibody, an anti-*E. coli* LPS primary antibody [Lipid A LPS Polyclonal Antibody (PA1-73178), Invitrogen] [38], diluted 1:2000 in 1% skim milk in TBS for 2 h. After five washes with TBS for 5 min each, the membrane was probed with the secondary antibody, an horseradish peroxidase (HRP)-conjugated donkey anti-goat IgG (H + L) antibody (A15999, Invitrogen), diluted 1:4000 in 1% (w/v) skim milk for 1 h. After five washes with TBS, detection was performed using the SuperSignal West Femto Maximum Sensitivity Substrate (Cat # 34095, Thermo Scientific) according to the manufacturer’s instructions.

### Statistical analysis

For each experiment, three independent experiments were performed on separate occasions, with the means and standard deviations presented in the graphs. Statistical analyses were conducted using the Student’s *t-*test. Statistically significant groups at *P* values of <.05, .01, .001, or .0001 are indicated within the figures (a, b, c, or d, respectively).

## Results and discussion

### Inner core LPS truncation broadly reduces susceptibility to *Bdellovibrio* predation

To define how LPS architecture influences prey susceptibility, we first examined predation against *E. coli* K-12 mutants carrying defined deletions in genes required for LPS core biosynthesis [28, 39, 40] (Fig. 1A). These experiments were conducted using four predator strains, including *B. bacteriovorus* 109J, HD100, BD-DH1, and EY2.3, which were selected as they represent both well-known strains (HD100 and 109J) as well as two relatively new isolates from South Korea (BD-DH1 and EY2.3) [9]. In each case, disruption of inner core biosynthesis in *E. coli* consistently reduced susceptibility to predation compared with the wild-type prey strain (Fig. S1). Tests confirmed these results were not due to differences in the bioluminescence outputs from these mutants (Fig. S2). Mutants lacking two inner core genes (*waaC* or *waaF*) or a gene involved outer core biosynthesis (*waaG*) showed a pronounced reduction in killing, whereas deletions affecting other outer core assembly genes (*waaB*, *waaI*, *waaJ*, or *waaL*) [12, 41, 42] produced weaker or predator-dependent effects (Fig. 1B and C). Under identical conditions, but without the predator, the prey viabilities were unaffected during these experiments, demonstrating the losses observed were due to the predators.

The inner core region of LPS is highly conserved among *Enterobacteriaceae* and contains heptose and Kdo residues that anchor the oligosaccharide to lipid A. Loss of these structures likely alters the physical and chemical properties of the outer membrane, and though they point toward the LPS as a potential receptor for *Bdellovibrio*, these changes may have broader impacts on outer membrane architecture and physiology, such as causing cell surface defects, to explain this phenomenon. The importance of the LPS, direct or indirect, however, is supported further by the results with the *rfaH* mutant (Fig. 1B and C), as deletion of this gene downregulates expression of multiple LPS biosynthetic genes and produces a truncated LPS structure [43, 44].

The reduced susceptibilities observed with the inner core mutants across the four predators tested suggests this region contributes to *Bdellovibrio* predation, although additional predatory strains and ecological contexts is needed to determine how broadly this phenotype is conserved. In ecological terms, this implies that variation in core LPS integrities among prey populations might strongly influence predator–prey dynamics within environments dominated by Gram-negative bacteria.

### Predator-specific susceptibility to outer core modifications reveals divergent predation patterns

Although inner core truncation reduced prey susceptibility to predation, the effects of specific LPS core modifications differed among the predators. For instance, HD100 and BD-DH1 showed reduced killing of mutants defective in *waaI*, which encodes an outer core glucosyltransferase involved in glucose addition to the outer core backbone, and *waaP*, which is required for phosphorylation of core heptose residues when compared with their individual activities against wild-type *E. coli* (Figs 1C, S1, and  S2). In contrast, EY2.3 was less efficient in predating mutants affecting the outer core structure (Δ*waaI*, Δ*waaJ*, and Δ*waaL*) [41, 42, 45] than it was against wild-type *E. coli*, whereas the activities of 109J against outer core mutants were largely unaffected (Figs S1 and S3). These results suggest an intact inner LPS core is broadly important for efficient predation by *Bdellovibrio* strains, although individual predators differ in their dependence on other LPS features. The basis for these differences, however, is unclear and may involve predator-specific interactions with additional prey surface structures, necessitating further work to clarify whether this reflects distinct prey recognition mechanisms or broader ecological specialization.

### Reduced susceptibility scales with the extent of LPS truncation but does not lead to resistance

The degree to which inner core truncation reduced susceptibility was also proportional to the severity of structural loss. Mutants producing the shortest LPS structures tended to show the greatest delay in predation (Fig. 1B) and the lowest frequencies of bdelloplast formation (*B. bacteriovorus* 109J; Fig. 1D) during early time points. Quantitative microscopy confirmed that *waaC*, *waaF*, or *waaG* mutant populations retained higher proportions of free, unattached prey cells compared with wild-type populations, but the loss of *waaI* or *waaL* had no apparent impact, with >94% of prey cells invaded for both of these mutants (Figs 1D and S4). Furthermore, viability losses (Fig. 1C) and bdelloplast populations (Fig. 1D) for all the mutants correlated well with each other (Fig. 1E; *R*^2^ = 0.84).

Despite truncation reducing susceptibility to predation, none of the LPS mutants were fully resistant to 109J or the other *B. bacteriovorus* strains. Low levels of bdelloplast formation were consistently observed across the mutant backgrounds (Fig. 1E). In long-term predation assays, the viability losses incurred by the mutants were comparable to those of wild-type *E. coli* BW25113 (Fig. S5). These observations indicate that LPS truncation delays, rather than prevents, predation, and suggest *Bdellovibrio* exploits secondary or lower-affinity interactions. From an ecological perspective, this also implies modifications in the LPS may act to reduce predation pressure without fully eliminating prey vulnerability, which would be evident as altered encounter rates and prey population dynamics rather than absolute resistance.

### LPS inner core integrity determines prey survival and susceptibility to *B. bacteriovorus* 109J predation

We performed mixed prey predation assays using strains with different LPS inner core lengths to determine how variations in core architecture affect prey survival during predation (Fig. 2A). After 90 min of predation by 109J, the isogenic *ΔwaaC*, *ΔwaaF*, and *ΔwaaG* mutants survived better than wild-type BW25113. In contrast, loss of *waaL* offered no clear survival benefit (Fig. 2A). This strain was included as a negative control because *waaL* encodes an O-antigen ligase but its protein is obsolete because *E. coli* K-12 strains do not synthesize an O-antigen.

**Figure 2:**
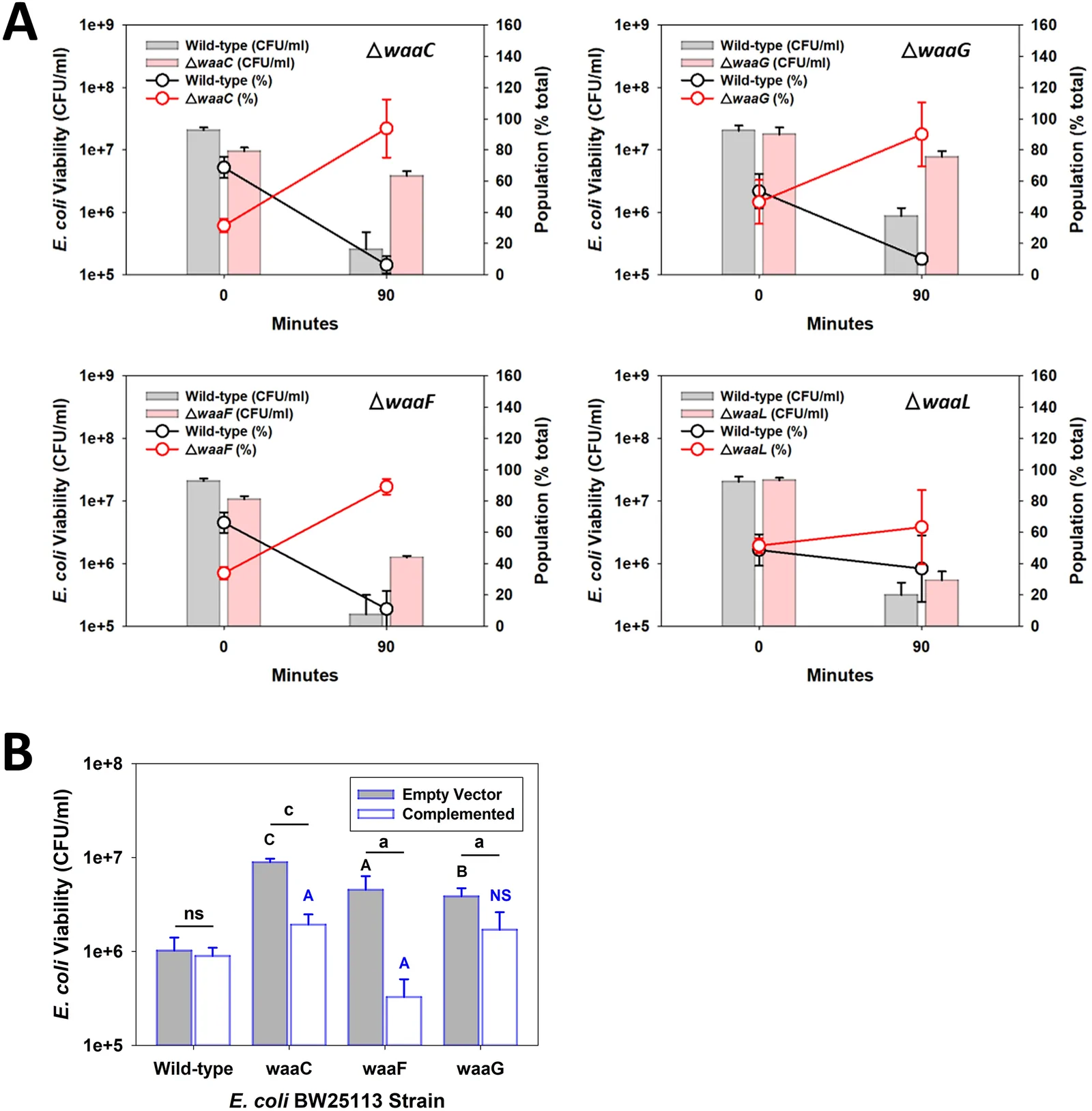
LPS core mutations affect prey survival during *B. bacteriovorus* 109J predation. (A) Mixed prey competition assays using wild-type *E. coli* BW25113 and the indicated LPS biosynthesis mutants, Δ*waaC*, Δ*waaF*, Δ*waaG*, or Δ*waaL*, during predation by *B. bacteriovorus* 109J. Prey viability was quantified by CFU enumeration at 0 and 90 min after co-incubation. Bars show CFU/ml for wild-type BW25113 and each mutant strain, corresponding to the left *y*-axis. Line plots show the relative proportion of each strain within the mixed prey population, corresponding to the right *y*-axis. (B) Complementation analysis of LPS inner core mutants. Wild-type BW25113 and Δ*waaC*, Δ*waaF*, or Δ*waaG* mutants carrying either the empty pCA24N vector or the corresponding complementation plasmid were exposed to *B. bacteriovorus* 109J, and prey viability was quantified by CFU enumeration. Different letters indicate statistically significant differences among groups, whereas ns or NS indicates no significant difference: a, *P <* .05; b, *P <* .01; c, *P <* .001; *n* = 3.

Because LPS inner core truncation increased relative prey survival, we tested whether complementation restores susceptibility to predation. Complementation of the individual inner core mutants (*ΔwaaC*, *ΔwaaF*, and *ΔwaaG*) increased their susceptibility to predation by 109J, with the 90-min viable counts approaching those of the wild-type strain (Fig. 2B). Considered alongside the mixed prey assays (Fig. 2A), these findings strongly indicate LPS inner core integrity has a significant impact on and influences prey survival during *B. bacteriovorus* 109J predation.

### Effects of exogenous LPS on 109J predation and prey-associated LPS dynamics

Given the strong influence of LPS architecture on susceptibility, the effects of exogenous LPS on predation by *B. bacteriovorus* 109J were examined. When *E. coli* K-12 LPS was added directly to predator–prey co-cultures, predation was somewhat delayed when compared with the untreated control (Fig. 3A). This effect was observed when LPS was present at the onset of predation, suggesting that free LPS interferes with predation under these conditions.

**Figure 3:**
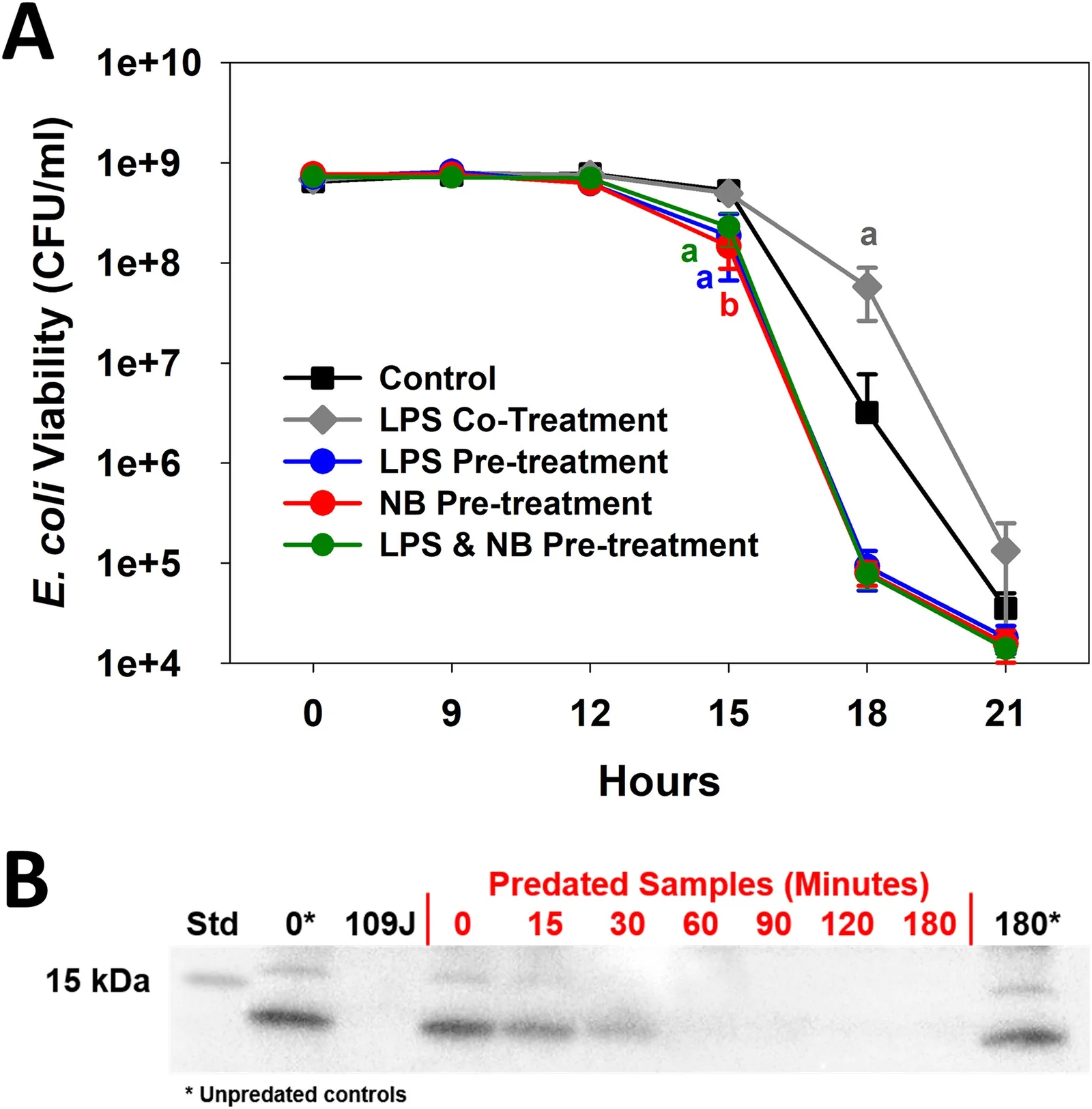
Effects of exogenous LPS and NB pretreatment on *B. bacteriovorus* 109J predation and prey LPS detection. (A) Viable *E. coli* prey counts during predation by *B. bacteriovorus* 109J under the indicated treatment conditions. Treatments included untreated 109J, LPS added at the start of predation, 109J pretreated with LPS for 1 h, 109J pretreated with NB for 1 h, and 109J pretreated with both LPS and NB for 1 h. Statistics compare treated samples with the untreated control: a, *P <* .05; b, *P <* .01; *n* = 3. (B) Western blot analysis of *E. coli* LPS during synchronized predation by 109J. Samples were collected at the indicated time points after initiation of predation. Std, LPS standard; 109J, predator-only control; asterisks indicate unpredated prey controls.

In contrast, pre-exposure of the predators to LPS accelerated predation (Figs 3A and S6). This effect was not due to a direct impact of LPS on prey viability, as LPS alone did not measurably affect *E. coli* BW25113 under the same conditions (Fig. S7). Pre-exposure to NB similarly enhanced predatory activity, whereas combined pre-exposure to LPS and NB did not further increase predation relative to either treatment alone. These results indicate exposure to exogenous LPS modulates the activity of attack-phase 109J similar to other nutrients.

Because LPS is a major component of the Gram-negative outer membrane, and extensive prey-envelope remodeling occurs during invasion and bdelloplast formation [46], we monitored LPS during a synchronized attack assay. Using Western blotting, we found the LPS signal decreased markedly within 0.5 h after predation began and continued to decline over time (Fig. 3B). This reduction indicates cellular LPS is progressively altered during predation by 109J, although whether this reflects degradation, envelope remodeling, or metabolic utilization remains unresolved. These results, however, may help explain previous studies that reported increased prey surface hydrophobicity during bdelloplast formation [47, 48].

### Restoring O-antigen biosynthesis partially reduces *E. coli* susceptibility

Because truncating the LPS reduces prey susceptibility to predation (Fig. 1), we examined whether extending the LPS polysaccharide also altered susceptibility. *Escherichia coli* K-12 strains, including BW25113, lack an O-antigen because they carry specific disruptive mutations within their *rfb* (O-antigen) gene cluster, most commonly an Insertion Sequence (IS5) element that inactivates the *wbbL* gene (encoding a rhamnose transferase) [49, 50]. To evaluate the potential impact of the O-antigen, therefore, the nonfunctional *wbbL* gene in *E. coli* BW25113 was complemented using plasmid pMF19 [36].

For 109J and BD-DH1, *wbbL* complementation significantly reduced killing efficiencies, whereas predation by HD100 or EY2.3 was unaffected (Fig. 4). To validate these results, *waaL* was deleted, as this would prevent ligation of O-antigen to the LPS core (Fig. 1A). This restored susceptibility to both 109J and BD-DH1, indicating the effects against the activities of these two predators require attachment of the O-antigen polysaccharide chains to the LPS molecule, rather than O-antigen synthesis alone.

**Figure 4:**
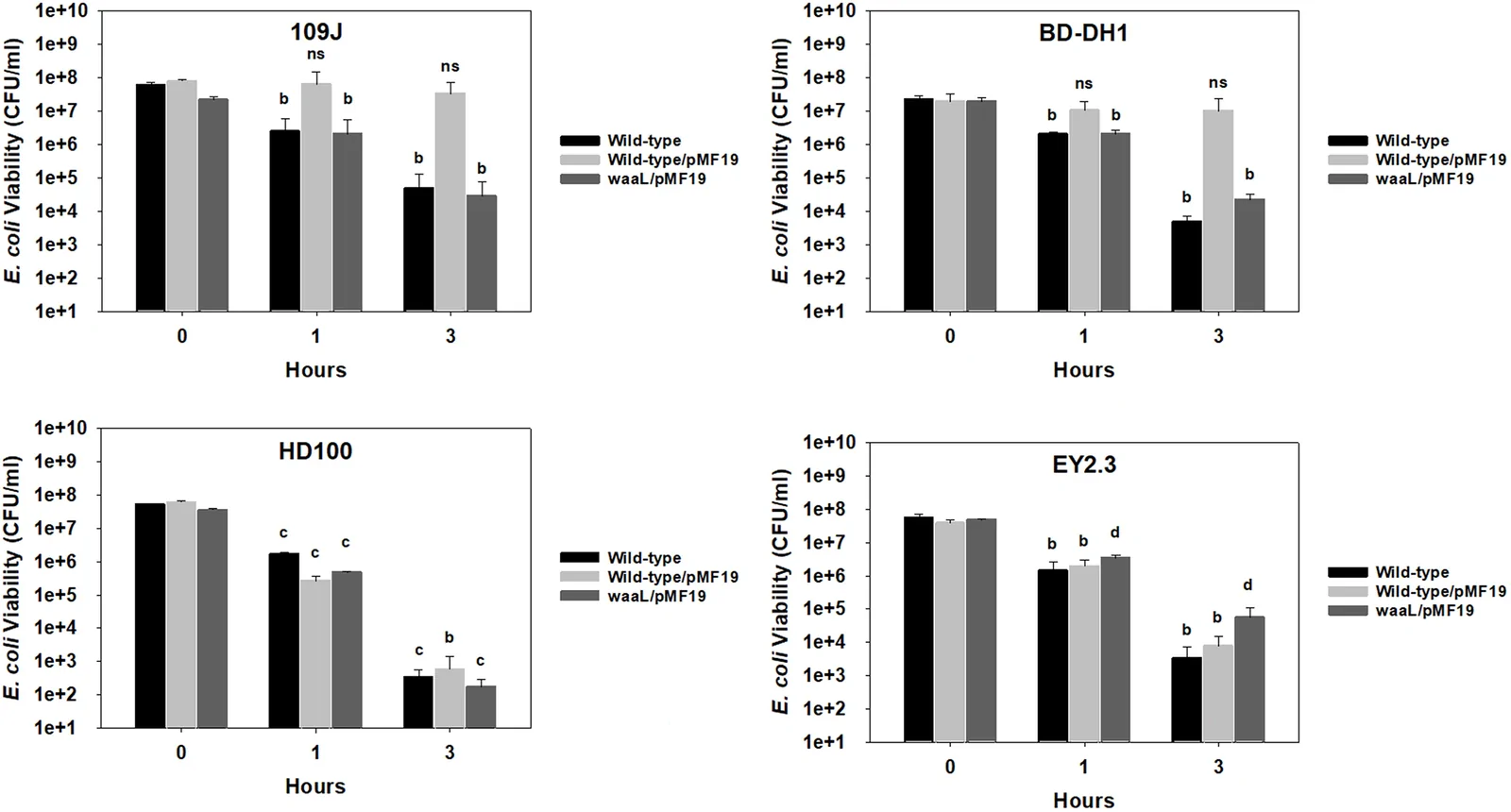
Effect of O-antigen restoration on predation of *E. coli* by different *Bdellovibrio* strains. Viable counts of *E. coli* BW25113 wild-type, BW25113 carrying pMF19 expressing *wbbL*, and the isogenic Δ*waaL* mutant carrying pMF19 during predation by (A) *B. bacteriovorus* 109J, (B) BD-DH1, (C) HD100, and (D) EY2.3. Prey viability was quantified by CFU enumeration at 0, 1, and 3 h after initiation of predation. The PPR for each sample was ~8.5. Statistics compare predated samples with the corresponding unpredated controls: ns, not significantly different; b, *P <* .01; c, *P <* .001; d, *P <* .0001; *n* = 3.

When covalently linked to the LPS core, O-antigen forms a dense, outward-projecting polysaccharide layer that can physically mask underlying surface structures. Comparable shielding effects have been widely documented in bacteriophage–host interactions [14–18] and in immune evasion by pathogenic bacteria [20], supporting the idea that extended surface polysaccharides can act as a general physical barrier to biological antagonists. The reduced susceptibility of O-antigen–restored strains to 109J and BD-DH1 is therefore consistent with the possibility that O-antigen alters the accessibility of LPS-associated surface features or adjacent outer membrane components involved in predation. In contrast, efficient predation by HD100 and EY2.3 following O-antigen restoration indicates steric exclusion is not a universal mechanism governing susceptibility to *Bdellovibrio* predation. Instead, these strain-specific differences suggest some predators engage alternative surface features that remain accessible despite O-antigen shielding, or that they possess attachment or invasion mechanisms that are capable of penetrating or bypassing extended polysaccharide layers, such as polysaccharide depolymerases [51]. HD100 remains sensitive to LPS truncation mutations, indicating that, although it tolerates O-antigen extension of the LPS, it nonetheless depends on aspects of intact LPS architecture for efficient predation.

### Goldilocks LPS architecture constrains predator–prey interactions across ecological scales

Taken together, the results presented in this study reveal prey susceptibility to *Bdellovibrio* predation is influenced by a constrained range of LPS architectures that balance structural integrity with surface accessibility (Fig. 5). Truncation of the LPS inner core reduces susceptibility by altering conserved core-associated features important for predator attachment and bdelloplast formation, whereas O-antigen extension reduces prey susceptibility to some predators, possibly by limiting access to underlying envelope structures. Efficient predation, therefore, appears to favor an LPS configuration that preserves core-associated features. Given the LPS inner core (Kdo/heptose region) is generally conserved across many Gram-negative taxa [12], this model may have relevance beyond *E. coli*, although additional prey species should be examined to determine its broader applicability.

**Figure 5:**
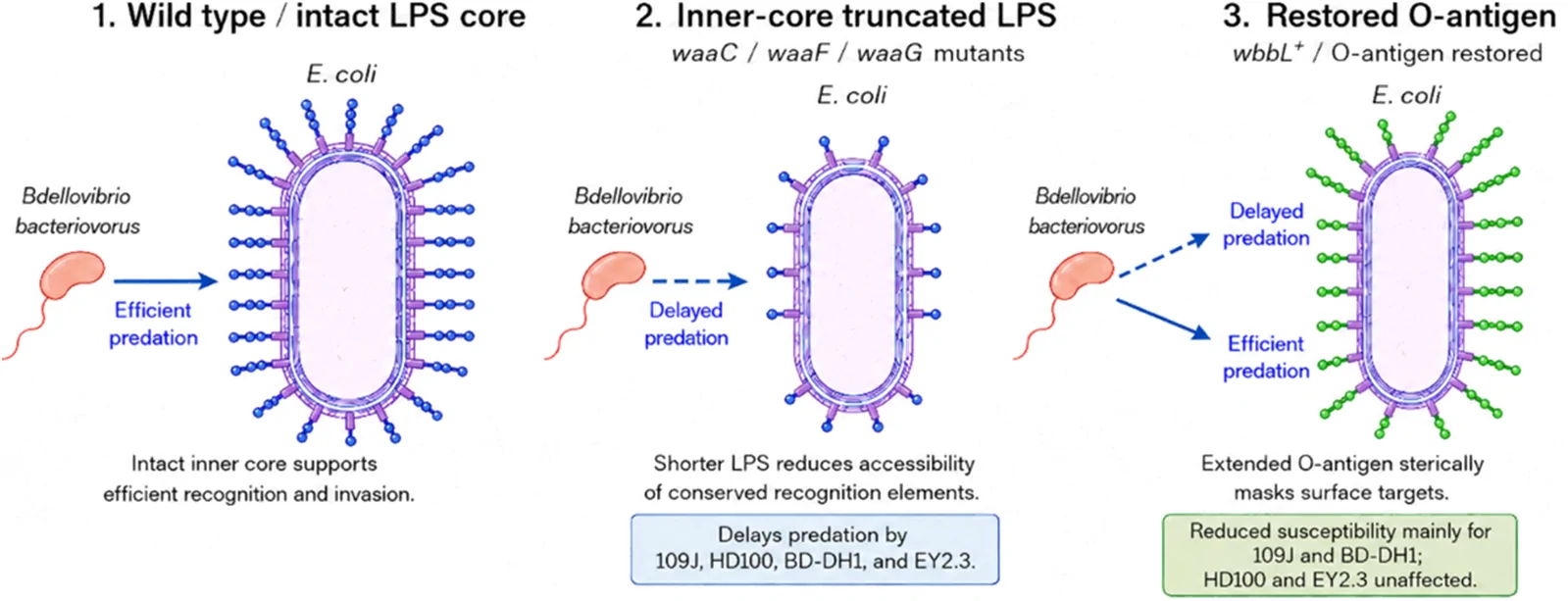
Schematic showing LPS architectural influences on *Bdellovibrio* predation. Schematic summary showing the three prey-envelope states examined in this study: wild-type *E. coli* with an intact LPS core, inner core truncated mutants, and strains with restored O-antigen production.

The LPS susceptibility window is not uniform across the predators tested, however, and may differ even further with other *Bdellovibrio* or BALO species. The distinct responses of the four predatory strains tested in this study to both LPS truncation and extension (Figs 1A and 4), however, suggest these four strains rely on partially overlapping yet nonidentical surface features during prey engagement. This predator-dependent susceptibility to prey surface architectures likely contributes to the observed variations in prey susceptibility among different *Bdellovibrio* strains [52], particularly in microbial communities where Gram-negative bacterial prey may coexist and display diverse envelope structures.

Rather than identifying a single optimal prey surface phenotype, however, the findings presented here indicate prey susceptibility is shaped by both its LPS architecture and the predator-specific responses to it. More broadly, however, BALO prey engagement is unlikely to depend on LPS architecture alone. This aligns with several previous studies that have shown *Bdellovibrio* interact with a variety of prey envelope-associated features during the early stages of predation [53], including outer membrane proteins [9] and surface glycans [54].

From the prey perspective, modulation of its LPS architecture provides a means to reduce, but not eliminate, predation pressure. This was observed here with both truncation and extension of the LPS molecule, but neither of these modifications fully prevented prey killing when incubations were extended. Consequently, LPS variations appear to act as a partial, quantitative modifier of prey susceptibility rather than as a binary barrier leading to complete resistance. In natural environments, prey LPS structures are influenced by several factors, including nutrient availability [55, 56], stress responses [57, 58], and selective pressures imposed by phage and host defenses [16, 59]. Such environmentally driven variations may therefore contribute to heterogeneity and selective pressure in Gram-negative bacterial populations when exposed to predatory bacteria. This was demonstrated here in the competition assays, where the relative population of survivors always favored the LPS-truncated genotypes (Fig. 2A).

Beyond its structural role in prey recognition or attachment, the findings of this study also suggest that extracellular or prey-associated LPS influence *Bdellovibrio* predation as pre-exposures to purified LPS enhanced prey killing rates with 109J (Fig. 3A) and prey-associated LPS signals decreased during synchronized predation (Fig. 3B). Such effects may be particularly relevant in prey-rich or biofilm-associated environments, where prey-derived envelope materials may accumulate, with further studies are needed to explore the ecological significance of extracellular LPS on *Bdellovibrio* strains in these settings.

Collectively, these findings establish prey LPS architectures as a central determinant of susceptibility to *B. bacteriovorus*. Efficient predation is optimized within a relatively narrow Goldilocks window of LPS configurations, where an intact inner core is preserved as excessive surface shielding by O-antigen extensions is avoided. This architectural selectivity varied across the predator strains tested, revealing divergent strategies among the four *Bdellovibrio* strains studied here. Beyond these immediate mechanistic insights, our results also suggest natural variations in LPS structure can generate heterogeneity in predation vulnerability within Gram-negative communities. Such dynamics likely shape predator–prey outcomes in complex microbiomes and, as such, have practical implications for the development of BALO-based biocontrol strategies. Future work targeting specific LPS–predator interactions or engineering prey surface features could further refine and deepen our understanding of bacterial predation and its impacts on microbial ecology.

## Supplementary Material

Supplemental_Information_wrag239

## Data Availability

All data are available in the main text or the supplementary materials. The predatory bacterial strains used in this study are available from the corresponding author upon request, subject to institutional approval and completion of an appropriate material transfer agreement.
