# Supplementary material for: Lipopolysaccharide architecture dictates susceptibility to predation by Bdellovibrio bacteriovorus
Source: ISME J. 2026 Sep 14;20(1):wrag239. doi: 10.1093/ismejo/wrag239 (PMC13768752; doi:10.1093/ismejo/wrag239)
Supplement: Supplementary file 1 [file supplemental_information_wrag239.docx]

**Supplemental Information**

**for**

**Lipopolysaccharide Architecture Dictates Susceptibility to Predation by *Bdellovibrio bacteriovorus***

Hyeon Seop Lee, Sumin Choi, Wonsik Mun^*^, and Robert J. Mitchell^*^

Department of Biological Sciences, Ulsan National Institute of Science and Technology, South Korea

^*^ - Corresponding Authors – WM ([wmun@unist.ac.kr](mailto:wmun@unist.ac.kr)) and RJM ([esgott@unist.ac.kr](mailto:esgott@unist.ac.kr))

**Supplementary Table S1. Bacterial strains and plasmids used in this study.** List of prey strains, predatory bacterial strains, and plasmids used in this study, including strain descriptions, relevant gene functions, antibiotic resistance markers, and source information.

| **Prey Strains** | **Description** | **Gene Function / Characteristics** | **References** |
| --- | --- | --- | --- |
| E. coli BW25113 | Wild-type strain | — | [1, 2] |
| E. coli JW3818 | Isogenic Δ*rfaH* | Transcription antiterminator | [1, 2] |
| E. coli JW3603 | Isogenic Δ*waaB* | LPS 1,6-galactosyltransferase | [1, 2] |
| E. coli JW3596 | Isogenic Δ*waaC* | LPS heptosyltransferase I | [1, 2] |
| E. coli JW3594 | Isogenic Δ*waaD* | ADP-L-glycero-D-manno-heptose-6-epimerase | [1, 2] |
| E. coli JW3024 | Isogenic Δ*waaE* | ADP-heptose-7-phosphate kinase | [1, 2] |
| E. coli JW3595 | Isogenic Δ*waaF* | ADP-heptose-LPS heptosyltransferase II | [1, 2] |
| E. coli JW3606 | Isogenic Δ*waaG* | glucosyltransferase I | [1, 2] |
| E. coli JW3590 | Isogenic Δw*aaH* | LPS glycosyltransferase | [1, 2] |
| E. coli JW3602 | Isogenic Δ*waaI* | UDP-galactose:LPS galactosyltransferase | [1, 2] |
| E. coli JW3601 | Isogenic Δ*waaJ* | LPS 1,2-glucosyltransferase | [1, 2] |
| E. coli JW3597 | Isogenic Δ*waaL* | O-antigen ligase | [1, 2] |
| E. coli JW3605 | Isogenic Δ*waaP* | LPS core heptose(I) kinase | [1, 2] |
| E. coli JW3607 | Isogenic Δ*waaQ* | LPS heptosyltransferase 3 | [1, 2] |
| E. coli JW3604 | Isogenic Δ*waaS* | LPS core biosynthesis protein | [1, 2] |
| E. coli JW3600 | Isogenic Δ*waaY* | LPS core heptose(I) kinase | [1, 2] |
| E. coli JW3598 | Isogenic Δ*waaZ* | LPS core biosynthesis protein | [1, 2] |
| **Predatory Strains** |  |  |  |
| B. bacteriovorus 109J | Bacterial predator | Sewage isolate from USA | ATCC 43826 |
| B. bacteriovorus HD100 | Bacterial predator | Soil isolate from California, USA | ATCC 15356 |
| Bdellovibrio sp. EY2.3 | Bacterial predator | Wastewater isolate from South Korean | [3] |
| Bdellovibrio sp. DH1 | Bacterial predator | Soil isolate from South Korean | [3] |
| **Plasmids** |  |  |  |
| pGEN-luxCDABE | Plasmid | Expresses *luxABCDE* (Amp 100 μg/mL) | [3-5] |
| pMQ414 | Plasmid | Expresses tdTomato mRFP gene (Gent 10 μg/mL) | [5, 6] |
| pMF19 | Plasmid | Expresses functional *wbbL* gene (Spc 50 μg/mL) | [7] |

**\**

**Supplementary Figure S1. Predation of E. coli LPS biosynthesis mutants by different Bdellovibrio strains.** Relative bioluminescence of wild-type E. coli BW25113 and the indicated LPS biosynthesis mutants following co-incubation with B. bacteriovorus 109J, HD100, BD-DH1, or EY2.3 for 1.5 hours. The mutations affecting LPS core biosynthesis and related envelope functions are indicated on the x-axis. Data are shown as mean ± SD. Letters indicate statistically significant differences relative to wild-type BW25113: a, *P* < 0.05; b, *P* < 0.01; c, *P* < 0.001; n = 3.

**Supplementary Figure S2. Bioluminescence values from the unpredated prey controls. The results showing similar outputs from each mutant across the predation time (90 minutes) used in Figure 1B.**

**Supplementary Figure S3. LPS biosynthesis mutations increase prey survival during predation by different Bdellovibrio strains.** Relative viability of E. coli BW25113 wild-type (BW) and the indicated LPS biosynthesis mutants following 90 min of predation by B. bacteriovorus 109J, HD100, BD-DH1, or EY2.3. Viable prey populations were determined by CFU enumeration, and survival is expressed as fold viability relative to the corresponding wild-type BW25113 population, which was normalized to 1 for each predator. Statistical comparisons were performed between the wild-type strain and each isogenic mutant: a, P < 0.05; b, P < 0.01; c, P < 0.001; n = 3.

**Figure S4. Representative confocal microscopy images of *E. coli* LPS mutants during predation by fluorescent *B. bacteriovorus* 109J.** Representative confocal microscopy images of wild-type *E. coli* BW25113 and the indicated LPS mutants after co-incubation with tdTomato-expressing *B. bacteriovorus* 109J. Images show brightfield prey-cell morphology overlaid with the red fluorescent predator signal. Scale bars, 5 µm.

**Supplementary Figure S5. Viability of E. coli LPS mutants after extended incubation with B. bacteriovorus 109J.** Initial and 24 h viable counts of wild-type E. coli BW25113 and the indicated LPS mutants during incubation with B. bacteriovorus 109J. Black circles indicate initial prey viability, and blue bars indicate prey viability after 24 h. Data are shown as mean ± SD.

**Supplementary Figure S6. Optical density measurements during B. bacteriovorus 109J predation under LPS and nutrient broth treatment conditions.** Changes in culture optical density during predation of E. coli by B. bacteriovorus 109J under the indicated treatment conditions. Treatments included untreated control, LPS co-treatment, 1 h LPS pre-treatment, 1 h nutrient broth (NB) pre-treatment, and 1 h LPS plus NB pre-treatment. Measurements were taken at 0, 9, 12, 15, 18, and 21 h after initiation of predation. Data are shown as mean ± SD. Letters indicate statistically significant differences between treated samples and the untreated control at the indicated time points: a, *P* < 0.05; b, *P* < 0.01; n = 3.

**Supplementary Figure S7. Viability of E. coli BW25113 following exposure to exogenous LPS.** Viable counts of E. coli BW25113 incubated with or without exogenous LPS (200 µg/ml) for 24 h. Prey viability was quantified by CFU enumeration at 0 and 24 h. Data are shown as mean ± SD. ns, not significantly different; n = 3.

**References**

E1.	Baba T, Ara T, Hasegawa M *et al.* Construction of *Escherichia coli* K-12 in-frame, single-gene knockout mutants: The KEIO collection. *Mol Syst Biol*. 2006;**2**:2006 0008 <https://doi.org/10.1038/msb4100050>

2.	Datsenko KA, Wanner BL. One-step inactivation of chromosomal genes in *Escherichia coli* K-12 using PCR products. *Proc Natl Acad Sci U S A*. 2000;**97**:6640–5 <https://doi.org/10.1073/pnas.120163297>

3.	Mun W, Upatissa S, Lim S *et al.* Outer membrane porin F in *E. coli* is critical for effective predation by *Bdellovibrio*. *Microbiol Spectr*. 2022;**10**:e0309422 <https://doi.org/10.1128/spectrum.03094-22>

4.	Lane MC, Alteri CJ, Smith SN *et al.* Expression of flagella is coincident with uropathogenic *Escherichia coli* ascension to the upper urinary tract. *Proc Natl Acad Sci U S A*. 2007;**104**:16669–74 <https://doi.org/10.1073/pnas.0607898104>

5.	Mun W, Choi S, Jang H *et al.* One-pot fermenter processing for the mass cultivation of predatory bacteria. *Chem Eng J*. 2024;**480**:148054 <https://doi.org/10.1016/j.cej.2023.148054>

6.	Mukherjee S, Brothers KM, Shanks RMQ *et al.* Visualizing *Bdellovibrio* *bacteriovorus* by using the tdtomato fluorescent protein. *Appl Environ Microbiol*. 2016;**82**:1653–61 <https://doi.org/10.1128/aem.03611-15>

7.	Feldman MF, Marolda CL, Monteiro MA *et al.* The activity of a putative polyisoprenol-linked sugar translocase (Wzx) involved in *Escherichia coli* O antigen assembly is independent of the chemical structure of the O repeat. *J Biol Chem*. 1999;**274**:35129–38 <https://doi.org/10.1074/jbc.274.49.35129>
